# A *Drosophila* Circuit Feels the (Sleep) Pressure

**DOI:** 10.1016/j.neuron.2014.02.002

**Published:** 2014-02-19

**Authors:** Güliz Gürel Özcan, Jason Rihel

**Affiliations:** 1 Department of Cell and Developmental Biology, University College London, Anatomy Building, Gower Street, London WC1E 6BT, UK

## Abstract

How sleep is homeostatically regulated remains a mystery. In this issue of *Neuron*, Donlea et al. (2014) provide evidence in *Drosophila* that a set of sleep-inducing neurons require Crossveinless-c, a specific Rho-GTPase-activating protein (Rho-Gap), to alter their membrane excitability in response to sleep deprivation.

## Main Text

The influential two-process model of sleep regulation posits that sleep pressure (i.e., the internal drive to sleep) is regulated by the interaction of circadian and homeostatic processes (Borbély, 1982). In this model, the circadian process synchronizes sleep drive to the 24 hr day-night cycle, while the homeostatic process steadily builds sleep pressure in response to wakefulness, then dissipates this pressure during sleep. Normally working in concert, the homeostatic process can be decoupled from the circadian process by sleep deprivation; as wakefulness is extended beyond normal physiological amounts, sleep pressure will also continue to build until it is homeostatically “reset” by subsequent rebound sleep. Although the mechanisms for coupling the circadian process to downstream sleep output remain murky, work in *Drosophila* and rodents over the past 40 years has painted a detailed picture of both the core molecular machinery (e.g., interlocking feedback loops among circadian clock proteins) as well as the critical pacemaker neurons (e.g., lateral neurons in *Drosophila*, the suprachiasmatic nucleus in mammals). Meanwhile, the homeostatic regulation of sleep is still shrouded in mystery. What aspects of prolonged waking drive sleep need? What are the molecular substrates by which this signal is transmitted? Where in the brain do these signals work to drive changes in sleep behavior?

Some progress has been made in identifying critical sleep-wake circuits. In the mammalian hypothalamus, sleep-active GABAergic neurons of the ventrolateral preoptic area (VLPO) form reciprocal inhibitory connections with a diverse set of wake-promoting neurons, known as the ascending arousal system (Saper et al., 2010). These circuits are considered critical drivers of sleep and wake, as ablation of the VLPO in rodents leads to insomnia, while pharmacological or optogenetic activation of components of the ascending arousal system promote waking (Rihel and Schier, 2013). An analogous sleep-wake circuit has recently been discovered in *Drosophila*. When directly activated by temperature-sensitive Trp channels, a set of neurons that project to the dorsal fan-shaped body (FB) induce sleep (Donlea et al., 2011). These neurons are directly connected to and inhibited by wake-promoting, FB-projecting dopaminergic neurons via the dopamine receptor DopR. Curiously, both the mammalian VLPO and the *Drosophila* FB sleep neurons are sensitive to the anesthetic isoflurane, and, at least in flies, this sensitivity is increased with sleep deprivation (Rihel and Schier, 2013). Given the central role that these neurons play as drivers of sleep/wake behavior, a natural hypothesis is that they will ultimately be sensitive, directly or indirectly, to the signal(s) of homeostatic sleep pressure. In this issue of *Neuron*, Donlea et al. (2014) push this hypothesis to the fore in *Drosophila*, arriving at an attractive albeit skeletal model whereby the electrical excitability of FB sleep output neurons is modulated in response to sleep deprivation.

In order to identify and manipulate the FB neurons, the previous studies relied on the same set of selective Gal4-driver lines. To start, Donlea et al. (2014) make the simple but excellent deduction that the underlying genes whose enhancers/promoters are hijacked by the Gal4-drivers will also be restricted in expression, critical for the functioning of these neurons, and, therefore, good candidate regulators of sleep. The transposon of one of the FB-restricted lines maps to an intron of a gene encoding a Rho GTPase-activating protein (Rho-GAP), *crossveinless-c* (*cv-c*). Flies harboring various mutations in *cv-c* sleep less, but they have normal waking activity and normal arousal threshold responses to stimuli (unlike many short-sleeping mutants). They also have normal circadian locomotor activity. However, when the flies were sleep deprived for 12 hr, *cv-c* mutants failed to show homeostatic rebound sleep, indicating that *cv-c* mutants are unable to either sense or convert increased sleep pressure into recovery sleep. An alternative explanation for this result alone is that *cv-c* mutants are “superflies” that require less sleep. However, *cv-c* mutants show impairments in an olfactory memory task, a result consistent with the cognitive deficits associated with chronic sleep deprivation. One caveat to this interpretation is that memory impairment may be a direct result of lost Cv-c function instead of a consequence of sleep deprivation. To address this, forcing the flies to sleep, through either pharmacological or direct activation of sleep circuits, should restore normal memory function if it is indeed due to chronic short sleep. Regardless, given that selective rescue of Cv-c in a few neurons restores sleep and memory (see below), they are likely to have defective sleep homeostasis, not a lower sleep need.

The *cv-c* mutants are not the first *Drosophila* sleep mutant to be identified with defects in sleep homeostasis. Previous molecular components implicated in *Drosophila* sleep homeostasis include cyclic-AMP and CREB signaling, ERK signaling, Shaker potassium channels and its regulator, Sleepless, dopamine, octopomine, and serotonin signaling, circadian clock components, cyclinA and its regulators, and the ubiquitin ligase Cullin-3 and its adaptor, Insomniac (Bushey and Cirelli, 2011; Rogulja and Young, 2012; Pfeiffenberger and Allada, 2012). In addition to the specificity of the *cv-c* behavioral phenotype (e.g., they are not hyper- or hypoactive, unlike many of the dopamine and insomniac mutants), what distinguishes the *cv-c* mutant from most of these others is the high degree of neuronal specificity. Replacing or depleting Cv-c function selectively in the sleep-inducing FB neurons is sufficient to respectively rescue or exacerbate the sleep homeostatic defect of *cv-c* mutants. Furthermore, altering Cv-c function in adult FB neurons demonstrates the sleep defect is not of developmental origin. Taken together, these data point directly to the FB neurons—and functioning Cv-c within these neurons—as critical for proper sleep homeostasis.

To explore this idea further, Donlea et al. (2014) performed direct electrophysiological recordings of both wild-type and *cv-c* mutant FB neurons before, during, and after sleep deprivation. First, they found a critical role for Cv-c in maintaining electrical excitability of FB neurons under current-clamp recordings—most wild-type FB neurons were excited by depolarizing current, while most *cv-c* mutant neurons remained electrically silent with reduced input resistances (R_m_) and membrane time constants (τ_m_). Cv-c is not required in all neuron types, as olfactory projection neurons remain electrically normal in *cv-c* mutants. Most intriguingly, they observed that wild-type FB neurons increased their electrical excitability in sleep-deprived flies and returned to baseline excitability following recovery sleep. This sleep deprivation-dependent modulation required functional Cv-c, as *cv-c* mutant FB neurons failed to alter electrical excitability to prolonged wakefulness.

**Figure 1:**
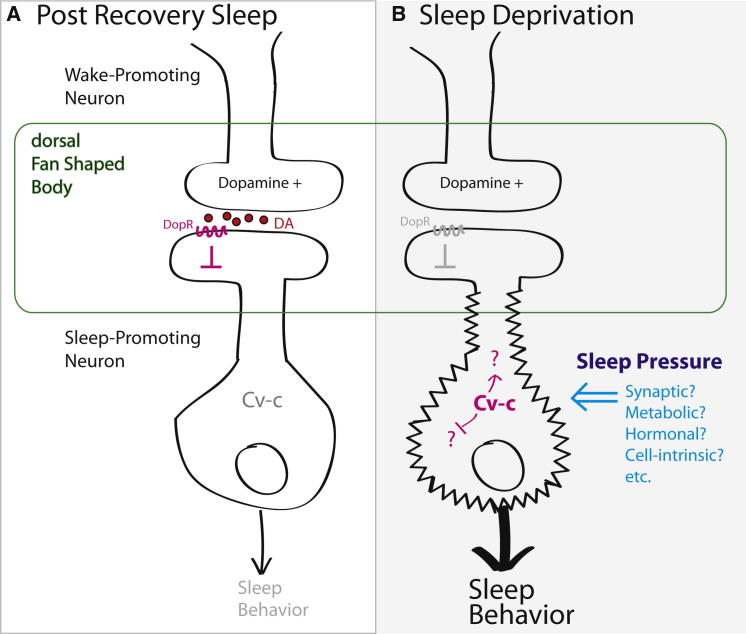
A *Drosophila* Sleep Pressure Point in the Fan-Shaped Body? (A) During low sleep pressure states, sleep-promoting FB neurons remain at their baseline excitability. (B) After sleep deprivation, sleep pressure signals are transmuted into increased electrical excitability of FB neurons in a process that requires functional Cv-c. The FB neurons are shown in context with wake-promoting dopamine neurons, which inhibit FB neurons and reduce sleep.

Only the scaffolding of a full sleep homeostasis model is brought into view by these results: some unknown direct or indirect signal for sleep pressure is transmitted into changes in electrical excitability of the major sleep output neurons, and this change depends, in an unknown way, on Cv-c (Figure 1). However, the potential implications of the model are substantial. In its strongest and perhaps most elegant form, the FB sleep output neurons themselves would act as a kind of sleep pressure antenna, directly receiving homeostatic cues and converting them into changes in electrical excitability, be these changes due to synaptic remodeling, metabolic cues, toxic breakdown products, hormonal signals of wakefulness, or even cell-intrinsic processes. Furthermore, how Cv-c might read sleep pressure signals and facilitate or convert this into electrical properties is unclear, although one potential clue may lie in Cv-c’s previously described role in synaptic homeostasis at the neuromuscular junction (Pilgram et al., 2011). Nevertheless, the model has the potential to unify myriad observations in *Drosophila* sleep studies. For example, Cv-c may regulate trafficking or channel properties of the sleep-relevant Shaker postassium channel or Sleepless within FB neurons, adding cellular specificity to these mutant phenotypes. Or perhaps Cv-c modulates cAMP/PKA signaling, which has been implicated in fly sleep homeostasis as well as dopamine inhibition of FB neurons. The model may also hint at possible mechanisms to explain other unusual observations. For example, starvation or methamphetamine sleep deprives flies without apparent rebound (Andretic et al., 2005; Bushey and Cirelli, 2011), perhaps because they prolong wakefulness yet short-circuit Cv-c-dependent electrical changes in FB neurons and block sleep homeostatic responses.

Finally, the question of whether the model may be directly relevant to mammals should be addressed. Does the analogy of the *Drosophila* sleep circuitry to the mammalian flip-flop circuit extend even to this nascent homeostasis model? One popular mammalian view envisions homeostatic sleep processes working on local cortical circuits (Krueger et al., 2008), in part because local slow waves respond homeostatically to use-dependent changes in neuronal activity. This view also reflects another distinction between mammalian and fly sleep rebound; namely, in mammals, sleep pressure more consistently tracks with the depth of recovery than the amount of rebound sleep (Bushey and Cirelli, 2011). However, sleep depth changes in flies have been measured and could also be regulated by the proposed homeostatic model (van Alphen et al., 2013). Furthermore, local mammalian sleep homeostats do not preclude the existence of additional, central mechanisms to relate sleep pressure to whole animal sleep. Indeed, a recent study found that the VLPO neurons increase their firing rate in response to sleep deprivation in a way that is sensitive to adenosine antagonism, one of the major metabolites suspected to signal sleep pressure in mammals (Alam et al., 2014).

To conclude, Donlea et al. (2014) have identified Cv-c as a molecular player in sleep homeostasis and more importantly have localized the effects to specific sleep-promoting cells in the *Drosophila* brain. Many questions remain, including whether the FB response to sleep deprivation also applies to the normal sleep-wake cycle, how sleep pressure is sensed by the FB cell, and how electrical excitability is restored following recovery sleep. While short on answers, the proposed model should now frame focused questions in *Drosophila* sleep research and should inspire the wider sleep community to investigate similar homeostatic models in a vertebrate context.
